# Quantitative image analysis of the extracellular matrix of esophageal squamous cell carcinoma and high grade dysplasia via two-photon microscopy

**DOI:** 10.1038/s41598-025-13910-7

**Published:** 2025-08-07

**Authors:** Kausalya Neelavara Makkithaya, Wei-Chung Chen, Chun-Chieh Wu, Ming-Chi Chen, Wei-Hsun Wang, Jackson Rodrigues, Ming-Tsang Wu, Nirmal Mazumder, I-Chen Wu, Guan-Yu Zhuo

**Affiliations:** 1 Department of Biophysics, Manipal School of Life Sciences, Manipal Academy of Higher Education, Manipal, 576104 India; 2 Division of Gastroenterology, Kaohsiung Medical University Hospital, Kaohsiung Medical University, Kaohsiung, 807 Taiwan; 3 Department of Pathology, Kaohsiung Medical University Hospital, Kaohsiung Medical University, Kaohsiung, 807 Taiwan; 4 Institute of Translational Medicine and New Drug Development, Medical University, Taichung, 404328 Taiwan; 5 Institute of Biophotonics, National Yang Ming Chiao Tung University, Taipei, 11221 Taiwan; 6 Department of Family Medicine, Kaohsiung Medical University Hospital, Kaohsiung Medical University, Kaohsiung, 807 Taiwan; 7 Center for Cancer Research, Center for Liquid Biopsy and Cohort Research, Kaohsiung Medical University, Kaohsiung, 807 Taiwan

**Keywords:** Esophageal cancer, Squamous cell carcinoma, Dysplasia, Two-photon microscopy, Second harmonic generation, Machine learning (ML), Gray-level co-occurrence matrix (GLCM), Support vector machine (SVM), Biophysics, Cancer, Diseases, Optics and photonics

## Abstract

Squamous cell carcinoma (SCC) and high-grade dysplasia (HGD) are two different pathological entities; however, they sometimes share similarities in histological structure depending on the context. Thus, distinguishing between the two may require careful examination by a pathologist and consideration of clinical findings. Unlike previous studies on cancer diagnosis using two-photon microscopy, quantitative analysis or machine learning (ML) algorithms need to be used to determine the subtle structural changes in images and the structural features that are statistically meaningful in cancer development. In this study, we aimed to quantitatively distinguish between SCC and HGD using two-photon microscopy combined with ML. Tissue samples were categorized into two groups: Group 1, primary SCC vs. metachronous HGD (SCC-HGD) and Group 2, primary HGD vs. metachronous HGD (HGD-HGD). We quantitatively analyzed second harmonic generation (SHG) and two-photon fluorescence (TPF) signals from two-photon microscopy imaging of the extracellular matrix (ECM). Gray-level co-occurrence matrix (GLCM) was used to extract the textural features of the tissue images, and support vector machine (SVM), for classification of the tissue images based on their pathologies. The SHG-based classifiers demonstrated 75%, 84.21%, 95%, and 95.65% for Group 1, Group 2, primary SCC vs. primary HGD, and metachronous HGD (Group 1) vs. metachronous HGD (Group 2), respectively. This integrative approach enabled the characterization of different pathological stages and enhances the understanding of the pathogenic factors involved in the progression of esophageal cancer.

**Supplementary Information:**

The online version contains supplementary material available at 10.1038/s41598-025-13910-7.

## Introduction

Esophageal cancer is one of the prevailing malignancies in several Asian countries, and its incidence rate is the 8th highest among cancers in the world^1^. The prognosis of esophageal cancer patients is poor, with a 5-year survival rate less than 20%^2^. This is attributed primarily to delayed disease diagnosis caused by the lack of early clinical indicators. Consequently, esophageal cancer often manifests at a later stage, significantly constraining the viable therapies available for effective treatment^3^. Because a considerable span of time required for normal cells and tissues to transform into invasive cancer cells or lesions, this time window can be used for the early detection of precancerous lesions, as well as for prohibiting or reversing the entire cascade of cancer inception, progression and metastasis. Despite the array of screening techniques designed to differentiate between benign, premalignant, and malignant lesions, various challenges hinder the effectiveness of these diagnostic approaches. Notably, the efficacy of clinical inspections for early cancer detection remains restricted due to the inconspicuous nature of certain lesions and the clinically normal-appearing mucosa that may harbor dysplasia or microinvasive carcinoma^4^.

Dysplasia has several key characteristics, including the presence of squamous cells exhibiting an atypical nuclear phenotype, disrupted cell polarity, and impaired maturation without penetrating the tissue basement membrane. Dysplasia is categorized based on the extent and distribution of abnormal cells within the epithelium. Low-grade dysplasia (LGD) is characterized by the confinement of abnormal cells to the lower third of the epithelium, while moderate dysplasia involves a greater extent of the epithelial layer. High-grade dysplasia (HGD) is characterized by the presence of abnormal cells extending to the upper third of the epithelium^5^. Carcinoma in situ (Cis) is considered equivalent to HGD because it involves the entire epithelium. Esophageal squamous cell neoplasms (ESCNs), which range from LGD to HGD/Cis and esophageal squamous cell carcinoma (ESCC), can occur in individuals with or without a history of head and neck squamous cell carcinoma (HNSCC). The emergence of secondary tumors in adjacent tissues may be elucidated by the theory of field cancerization, which posits that multiple cancers can originate from a single area of epithelium chronically exposed to carcinogenic factors^6^. Consequently, metachronous neoplasms (diagnosed by ESCNs more than six months later) are considered distinct, independent tumors under this framework^7^.

The gold standard for cancer diagnosis is histopathological examination or tissue biopsy^8^. Hematoxylin and eosin (H&E) staining is a routine and widely used technique in pathology that is employed in conjunction with tissue biopsy samples as part of histopathological examination^9^. However, H&E staining is limited to cell nuclei and does not effectively capture the complexities of intra- and extracellular tissue proteins; furthermore, interpreting the results of H&E-stained sections often relies on the subjective judgment of the observer. By utilizing H&E-stained sections for two-photon imaging, based on second harmonic generation (SHG) and two-photon fluorescence (TPF), the technique can be easily integrated into existing clinical workflows without requiring additional sample preparation and surpasses the limitations of H&E staining by offering complementary information to conventional histological analysis. While H&E staining primarily highlights cellular morphology and tissue architecture, SHG and TPF imaging can reveal additional details about collagen fibers in the extracellular matrix (ECM) and the presence of endogenous fluorophores in layered histopathological structures that encompass collagen, elastin, blood vessels, enzymes, and more, respectively^10–12^. This enhanced visualization can aid pathologists in identifying and characterizing morphological changes associated with cancer progression. Notably, changes in collagen organization and distribution are often associated with cancer development and progression. By imaging H&E-stained sections with SHG, clinicians can assess the integrity and alterations of the collagen network, potentially aiding in cancer diagnosis and staging^10,13–15^.

Two-photon imaging of H&E-stained sections generates high-resolution, digital images that can be analyzed using advanced image processing techniques and machine learning (ML) algorithms^16^. This opens up the possibility for automated analysis and quantification of tissue features, such as collagen fiber organization or cellular morphology, which could assist in an objective and reproducible cancer diagnosis. As the demand for precise and nuanced image analysis continues to grow, the utilization of advanced computational techniques becomes paramount. Within this context, gray level co-occurrence matrix (GLCM) feature extraction has emerged as a powerful method for quantifying and characterizing textural patterns in images^10,17,18^. Texture features hold significant importance in medical imaging due to their inherent structural simplicity. The GLCM technique is rooted in statistical analysis, which systematically explores the relationships between pixel intensities by considering the frequency of co-occurring pixel pairs at varying spatial displacements. This technique is used to extract textural features such as Contrast, Energy, Angular Second Moment (ASM), Correlation, Homogeneity, etc., from the input images. The GLCM is a matrix, with the number of columns and rows equivalent to the number of distinct pixel values within a specific image region. The GLCM not only defines the intensity relationship between two pixels but also represents the size, direction, and intensity level of a pixel with respect to its neighboring pixels. In addition, the support vector machine (SVM) is a supervised ML algorithm commonly used for handling high-dimensional data and classification tasks^19,20^. When the dataset is relatively small and a high demand for accuracy and generalization is needed, SVM is known to be particularly useful.

SCC and HGD in esophageal tissues exhibit a range of similar structural features, with HGD often considered a precursor lesion to SCC. While histopathological examination reliably distinguishes between SCC and HGD^21–23^differentiating among various instances of HGD poses a significant challenge for pathologists due to their similar appearances under microscopic examination. According to the theory of field cancerization, the emergence of secondary (metachronous) HGD is influenced by the same carcinogenic factors as the initial lesion. It is hypothesized that HGD derived from SCC may exhibit more severe tissue abnormalities than HGD originating from standalone, potentially serving as early indicators of subsequent dysplastic development. Although the current treatment protocols for both HGD and early-stage SCC typically involve endoscopic resection to excise abnormal tissues^24,25^there is a compelling case for adopting a more aggressive treatment approach for severe cases of HGD. This strategy aims to prevent further dysplastic changes and shorten the follow-up intervals to prevent abrupt progression. Furthermore, integrating two-photon microscopy with advanced ML algorithms holds promise for enhancing the precision of differentiating between SCC and HGD. This approach not only provides deeper insights into the early stages of cancer progression by analyzing structural parameters calculated through ML techniques but also lays the groundwork for developing predictive models to assess the likelihood of cancer relapse.

## Materials and methods

### Tissue information and slide preparation

In this study, twelve subjects were selected from an upper endoscopic surveillance cohort of patients with incident head and neck squamous cell carcinoma (HNSCC) at Kaohsiung Medical University Hospital between 2008 and 2018. The initial cohort comprised 1,042 patients. The inclusion criteria were newly diagnosed HNSCC and availability for endoscopic surveillance. The exclusion criteria included preexisting malignancies, prior esophageal surgery, total luminal obstruction by tumor, emergent surgery due to tumor-related complications, refusal or ineligibility for endoscopy, incomplete clinical data, synchronous esophageal neoplasia, single-time screening only, or non-squamous histology.

Among the 1,042 eligible patients, 47 developed primary esophageal HGD and 61 developed primary esophageal SCC. Of these, 9 HGD and 12 SCC cases later developed metachronous HGD. After reviewing the availability and histological quality of H&E-stained slides, three HGD and nine SCC cases with adequate slide quality were included in the final analysis—comprising Group 2 (n = 3) and Group 1 (n = 9), respectively.

A cohort selection diagram (Fig. S1 in the supplementary material) has been added to visually illustrate the stepwise inclusion and exclusion process. All study procedures were approved by the Kaohsiung Medical University Hospital Institutional Review Board (KMUH-IRB-980559). Informed consent was obtained from all participants, and demographic and lifestyle information was collected through structured interviews. All methods followed relevant institutional and international guidelines.

**Fig. 1:**
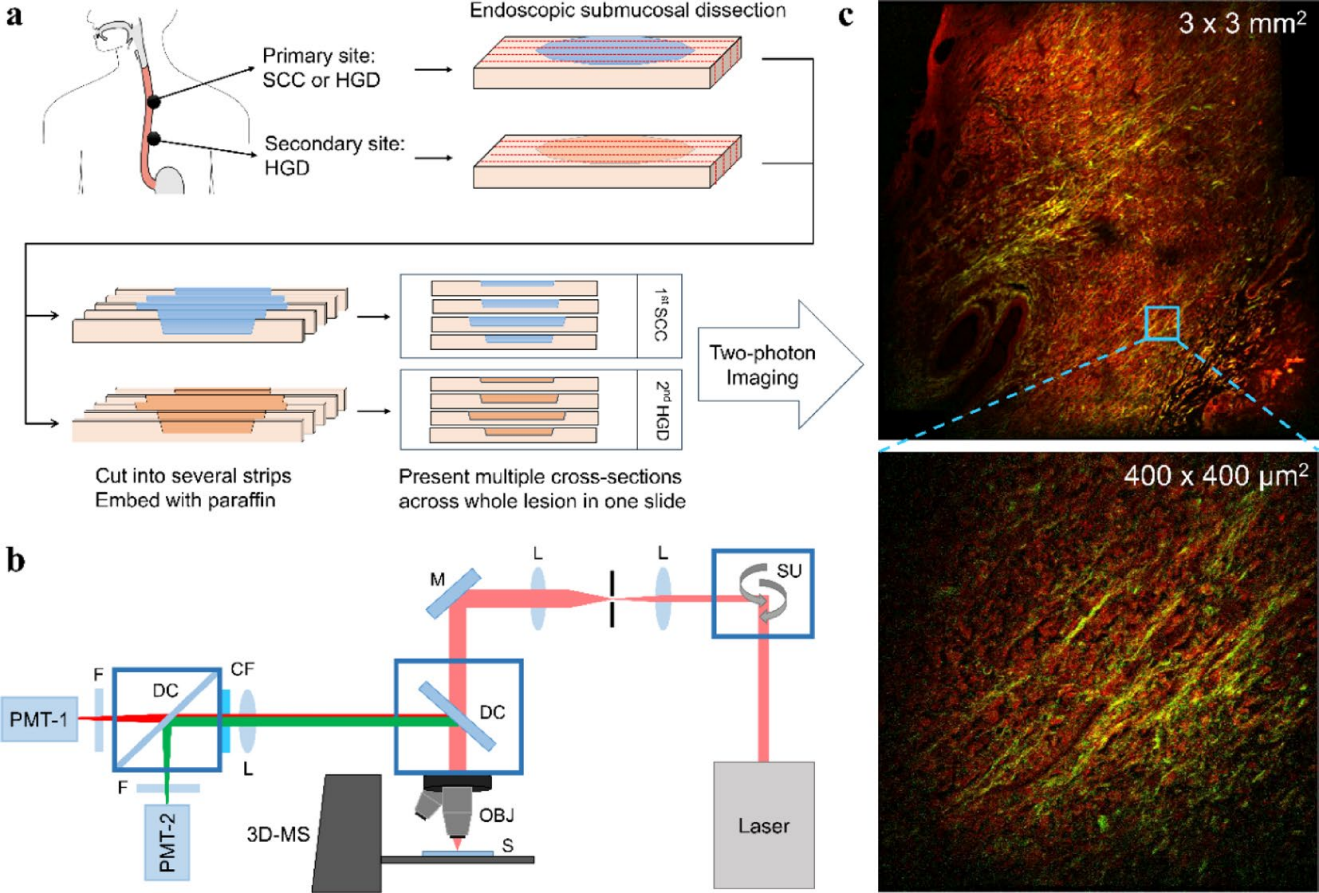
(a) Flowchart showing the processes from tissue slide preparation to histological analysis and two-photon image analysis. (b) The microscope setup for two-photon imaging. SU: scan unit; L: lens; M: mirror; DC: dichroic beamsplitter; OBJ: objective lens; S: sample; 3D-MS: three-dimensional motorized stage; CF: color filter; F: filter; PMT: photomultiplier tube. (c) The demonstration of a large-field image (top) tiled from single 2D images (bottom). The green‒yellow color indicates the SHG presenting collagen fibers, while the red color indicates the TPF presenting layered histopathological structures.

Figure 1(a) illustrates the tissue slide preparation process for lesion identification. The primary site tissue varied between SCC and HGD depending on the subject, while all secondary site tissues were HGD. Endoscopic submucosal dissection was performed to separate the diseased tissue from the submucosa. The resected tissues underwent processing, including cutting into 10 μm thick strips and paraffin embedding, for histological analysis under a white-light microscope. The paraffin-embedded tissue was then sectioned to create multiple cross-sections on a single slide for two-photon imaging analysis of SHG and TPF characteristics. During the screening process, biopsies were performed on all lesions larger than 1 cm and as many additional lesions as possible when multiple esophageal lesions were present. Patient classification was based on the most severe pathological finding from the screening. Each patient provided two slides: one for the original diagnosis and another for follow-up after 6 months. Large-scale images of each slide were obtained using an image stitching method at 6 different locations. While the sample size is limited, it reflects the rarity of these specific pathological conditions and the stringent quality control measures implemented in our study. The robustness of our analytical approach, combining two-photon microscopy with advanced image analysis techniques, helps mitigate this limitation by providing detailed quantitative characterization of ECM-specific changes in these carefully selected cases.

### Custom-built two-photon microscopy

A custom-built laser scanning microscope (Fig. 1(b))^26,27^ was developed to alleviate some of the disadvantages of traditional histopathology by using various endogenous nonlinear optical (NLO) signals to obtain virtual biopsies that present the architecture and composition of tissues. This microscopy system is composed of a femtosecond fiber laser (Kasmoro-780, mRadian, Taiwan) with the characteristics of 780 nm central wavelength, 300 fs pulse width and 450 mW total power, a galvanometer scanner assembly (GVS012, Thorlabs, USA), and a 3D motorized stage (PT3-Z8, Thorlabs, USA) that is synchronized with the scanning unit via the control and operation interface provided by Southport Ltd., Taiwan. The laser beam focusing and collection of emitted photons are performed by an air objective lens (UPlanSApo 20x/0.75, Olympus, Japan) in epi-detection geometry. To ensure the purity of the emitted photons and separate them into their respective channels, a set of filters (FF01-390/40 (for SHG detection) and FF01-488-50 (for TPF detection), Semrock, USA) and dichroic beamsplitters (DMLP425R (for separation of SHG and TPF) and DMLP735B (for separation of NLO signals and excitation laser), Thorlabs, USA) are used. Finally, the signals are detected by two identical photomultiplier tubes (R3896, Hamamatsu, Japan) for further signal digitization and image formation. Notably, repeated observations confirmed that no signal crosstalk from TPF to the SHG channel, as areas without collagen fibers showed no detectable signal in the SHG channel. In the experiment, first, esophageal tissue slides were subjected to ex vivo two-photon imaging, after which the results were compared with those of H&E staining identified by pathologists. Afterwards, the correct data were used to train the ML algorithm for subsequent AI-assisted pathology. For validation, we investigated the differences in tissue organization between primary esophageal HGD with metachronous HGD (Group 1) and primary ESCC with metachronous HGD (Group 2) and evaluated whether these differences can be used as diagnostic markers.

To achieve higher classification accuracy, the sample quantity must be increased as much as possible via possible methods. Combining image stitching with two-photon microscopy facilitates the acquisition of large-field images of esophageal cancer tissues to generate large datasets. Therefore, training with larger datasets and more clinical validations will be helpful for identifying whether other biomarkers play a crucial role in ESCN pathologies and can provide useful information for histopathologists to determine the onset of esophageal cancer. Regarding the process of image stitching, first, we used the microscope for two-channel imaging, as shown in Fig. 1(c), in which SHG images the collagen fibers shown in green‒yellow and TPF images layered histopathological structures shown in red. The image size of a single 2D image is 400 × 400 µm^2^ (pixel dwell time: 4 µs, for imaging 512 × 512 pixels). Then, the stage was synchronized and automatically translated step by step following imaging style requests. In this case, we imaged the sample as 15 × 15 images, totaling 225 images. Finally, we used Image/Fiji software (National Institutes of Health, USA) to stitch all the 2D images and to reconstruct a large-field image, with a total processing time approximately 15 minutes^28^.

### Texture analysis of GLCM and classification using SVM

Texture is a description of the local intensity change from pixel to pixel within a small neighborhood of a picture. Textural features capture information about the spatial arrangement of pixel intensities in an image by quantifying them, thus revealing patterns and structures that may not be immediately apparent through visual inspection^29,30^. GLCM algorithm was used in this study to systematically capture the textural features of the esophageal cancer images. This is achieved by the GLCM through the analysis of the co-occurrence of the pixels at different positions within the image. In the present study, textural feature extraction from whole images of the tissue samples was performed. The images were grouped according to the diagnoses and analyzed. First, the images were split into their respective channels, i.e., SHG and TPF channel images. The images were then converted into grayscale, thus standardizing the data for subsequent texture analysis. Textural features were extracted from these grayscale images using the GLCM algorithm. To ensure a robust and orientation-invariant assessment, features were calculated at four different angles: 0°, 45°, 90°, and 135°. This multi-angle approach allows for the detection of textural patterns regardless of their directional alignment within the tissue. The extracted features included commonly used GLCM-derived metrics such as contrast, correlation, energy, and homogeneity, which were subsequently used to train the SVM algorithm for classification of the diagnoses in the groups of images.

SVMs are well-suited for high-dimensional data and operate by identifying the optimal hyperplane that maximizes the margin between different diagnostic groups in the feature space^19^. In this study, a linear kernel was chosen for the SVM, as it offers interpretability and is effective when the classes are linearly separable in the transformed feature space. To rigorously evaluate the classification performance and mitigate the risk of bias due to class imbalance, we implemented stratified k-fold cross-validation. In this study, the data was split into nine folds by the stratified k-fold cross validation algorithm. This technique ensures that each fold of the data preserves the original class distribution, providing a more representative and reliable assessment of the model’s generalizability. Stratification is particularly important in biomedical datasets, where the prevalence of different diagnoses can vary substantially^31^. By maintaining proportional representation of each class in every fold, stratified cross-validation reduces the likelihood of overfitting to the majority class and yields more accurate estimates of the classifier’s performance across all diagnostic categories.

The algorithms for GLCM and SVM were written in python, importing libraries such as sklearn, numpy, matplotlib, and pandas. The following SVM models were trained to compare the classification of image features from the esophageal cancer groups: (a) primary SCC vs. metachronous HGD (b) primary HGD vs. metachronous HGD (c) primary SCC vs. primary HGD (d) metachronous HGD from SCC vs. metachronous HGD from HGD. Each model was trained with data from SHG and TPF images for further comparison of textural data obtained from esophageal cancer images of the two modalities. Furthermore, an area under the curve of the receiver operating characteristic (AUC-ROC) curve was plotted for each classifier^32,33^wherein the AUC-ROC curve was plotted for each fold in the respective models, and the fold with the best performance was highlighted. The corresponding confusion matrix for the best performing fold in the model was also plotted. The AUC-ROC curve is a graphical representation of the performance of a binary classification model at various classification thresholds. It is commonly used in ML to assess the ability of a model to distinguish between two classes, typically the positive class (e.g., presence of a disease) and the negative class (e.g., absence of a disease).

## Results

### Visualization of the morphological structure of esophageal tissues

**Fig. 2:**
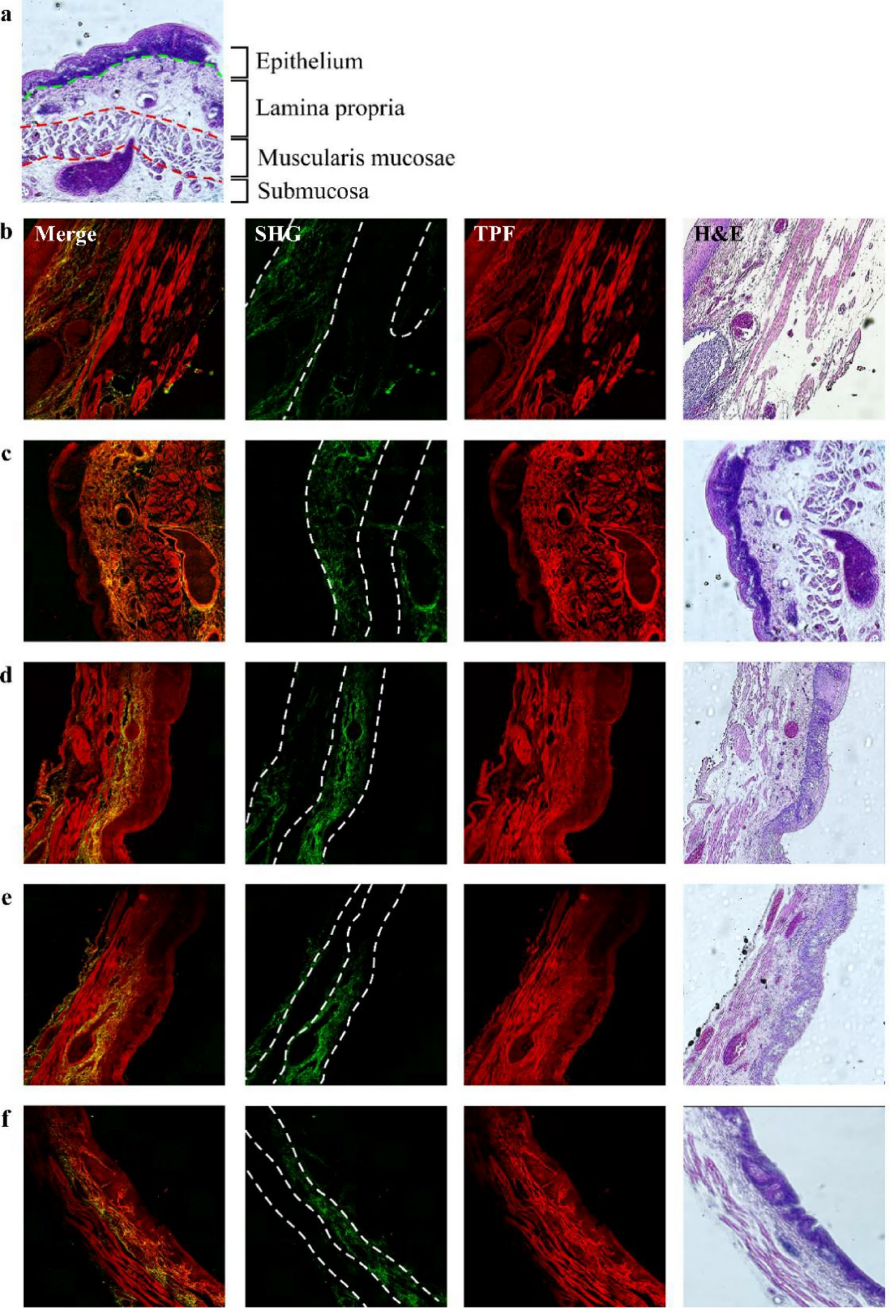
Representative two-photon images of esophageal cancer tissues from patients diagnosed with SCC at the first diagnosis. (a) H&E-stained histological image showing the layered structures of esophageal tissue. *(b)-(f) show five image sets. Merge indicates the overlaying of SHG and TPF images. Dashed lines represent layer boundaries.* Image size: 1.6 × 1.6 mm^2^.

In the human esophagus, which extends from the inner lumen to the outer adventitia, the tissue is stratified into several layers: the keratinized layer, mucosa, submucosa, muscularis propria, and adventitia. Our study specifically targets the early onset of high-grade dysplasia, which was dissected using endoscopic submucosal dissection and primarily encompasses the mucosa and submucosa tissues. The mucosal layer is further subdivided into three distinct zones: the epithelium, lamina propria (LP), and muscularis mucosae (MM), as illustrated in Fig. 2(a). The epithelial layer is characterized by squamous cells that proliferate and ascend, continuously renewing the epithelial lining. The lamina propria, a slender layer of connective tissue, bridges the epithelium with the smooth muscle cells of the muscularis mucosae. It provides an essential blood supply to the epithelium and neural connections that stimulate the underlying muscularis mucosae. The muscularis mucosae plays a crucial role in stretching and contracting the mucosa, aiding in the formation of a mucosal plug or a one-way valve during swallowing.

**Fig. 3:**
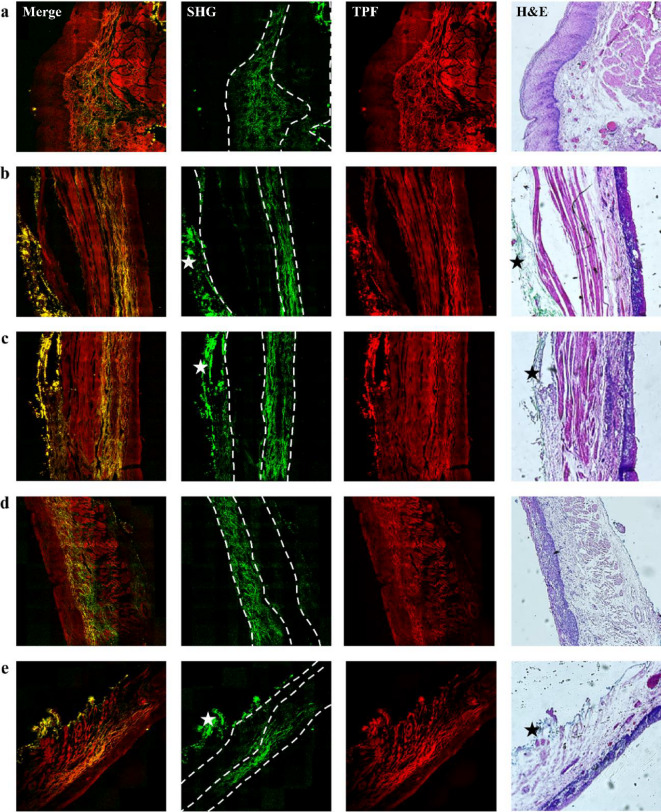
Representative two-photon images of esophageal cancer tissues from patients diagnosed with HGD for the second diagnosis (as the follow-up 6 months after the first diagnosis shown in Fig. 2). *(a)-(e) show five image sets. Merge indicates the overlaying of SHG and TPF images. Dashed lines represent layer boundaries*,* while stars indicate the artifacts presumably due to photodamage from dye accumulation.* Image size: 1.6 × 1.6 mm^2^.

**Fig. 4:**
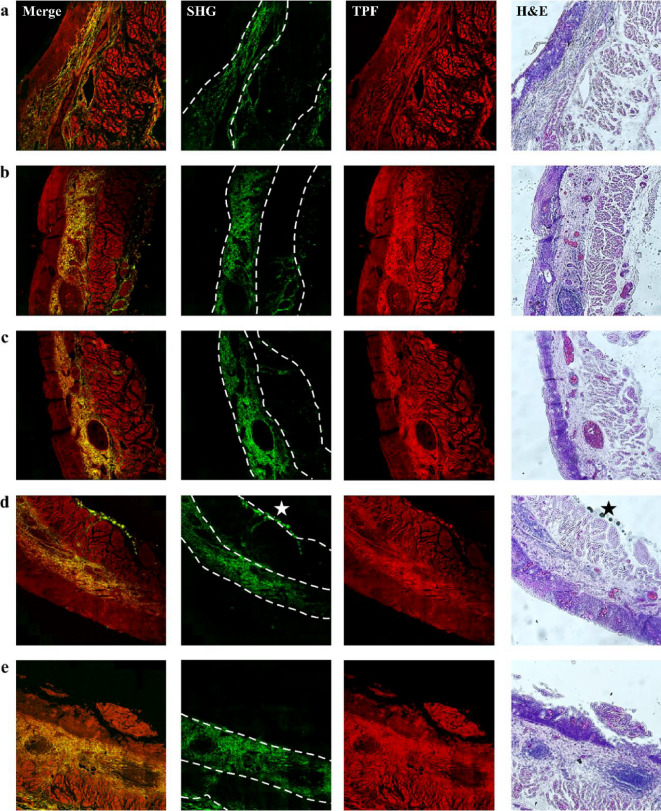
Representative two-photon images of esophageal cancer tissues from patients diagnosed with HGD for the first diagnosis. *(a)-(e) show five image sets. Merge indicates the overlaying of SHG and TPF images. Dashed lines represent layer boundaries*,* while stars indicate the artifacts presumably due to photodamage from dye accumulation.* Image size: 1.6 × 1.6 mm^2^.

**Fig. 5:**
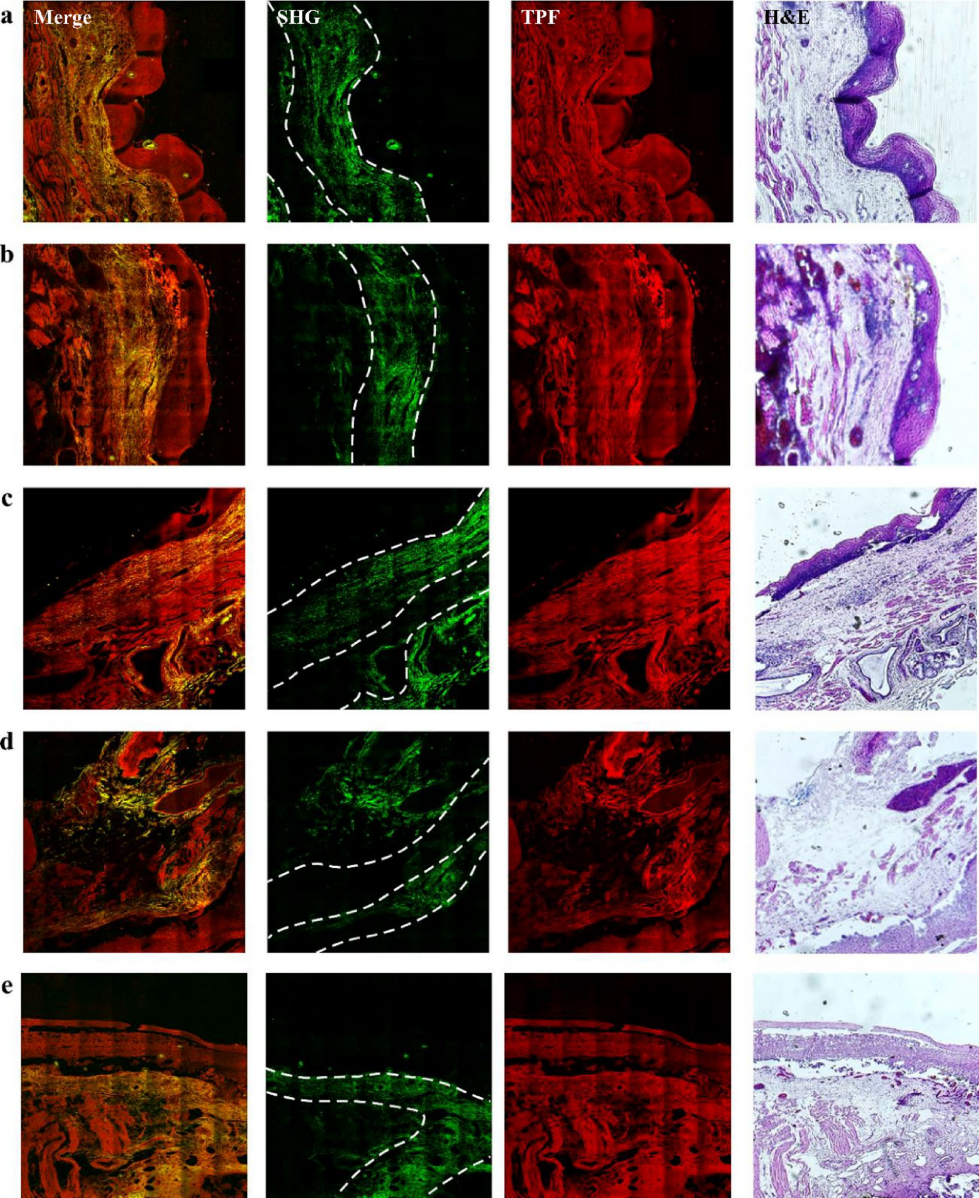
Representative two-photon images of esophageal cancer tissues from patients diagnosed with HGD for the second diagnosis (as the follow-up 6 months after the first diagnosis shown in Fig. 4). *(a)-(e) show five image sets. Merge indicates the overlaying of SHG and TPF images. Dashed lines represent layer boundaries.* Image size: 1.6 × 1.6 mm^2^.

Figures 2, 3 and 4, and Fig. 5 show that the collagen SHG signals predominantly appear in the connective tissues of the LP and submucosa but not in the epithelium or MM. For instance, in Fig. 2(c), the area between the left and middle white dashed lines represents the LP, which exhibits collagen signals, whereas the area between the middle and right dashed lines corresponds to the MM, which lacks collagen. The submucosal region, to the right of the right dashed line, exhibits strong collagen signals, akin to those in the LP region. As dysplasia originates in the epithelium and gradually evolves into squamous cell carcinoma invading the submucosa, alterations in collagen within the LP can yield crucial insights into the current state of the corresponding HGD or SCC above it. To ensure accurate GLCM analysis, we excluded artifact signals from dye accumulation at section edges. These artifacts produce broadband emission in both spectral channels presumably due to photodamage (marked by stars in Figs. 3(b), 3(c), 3(e), and 4(d)). From these images, we found that the tissue morphology all contained layer structures, as indicated by Fig. 2(a), and that the ECM contained partially oriented collagen fibers (shown by SHG signals) nearly parallel to the epithelium. As these characteristics are visually similar, it is difficult to distinguish between the four types of tissues. To reveal the structural differences embedded in these tissues, ML algorithms, as demonstrated in the following sections, were used.

### GLCM analysis based classification using SVM

In the current study, we investigated the different morphological features of collagen fibers in the ECM between primary HGD with metachronous HGD and primary SCC with metachronous HGD groups and evaluated whether these differences can be used as biomarkers for early cancer detection. Thus, the acquired images were analyzed using the GLCM method to quantitatively classify the samples.

**Fig. 6:**
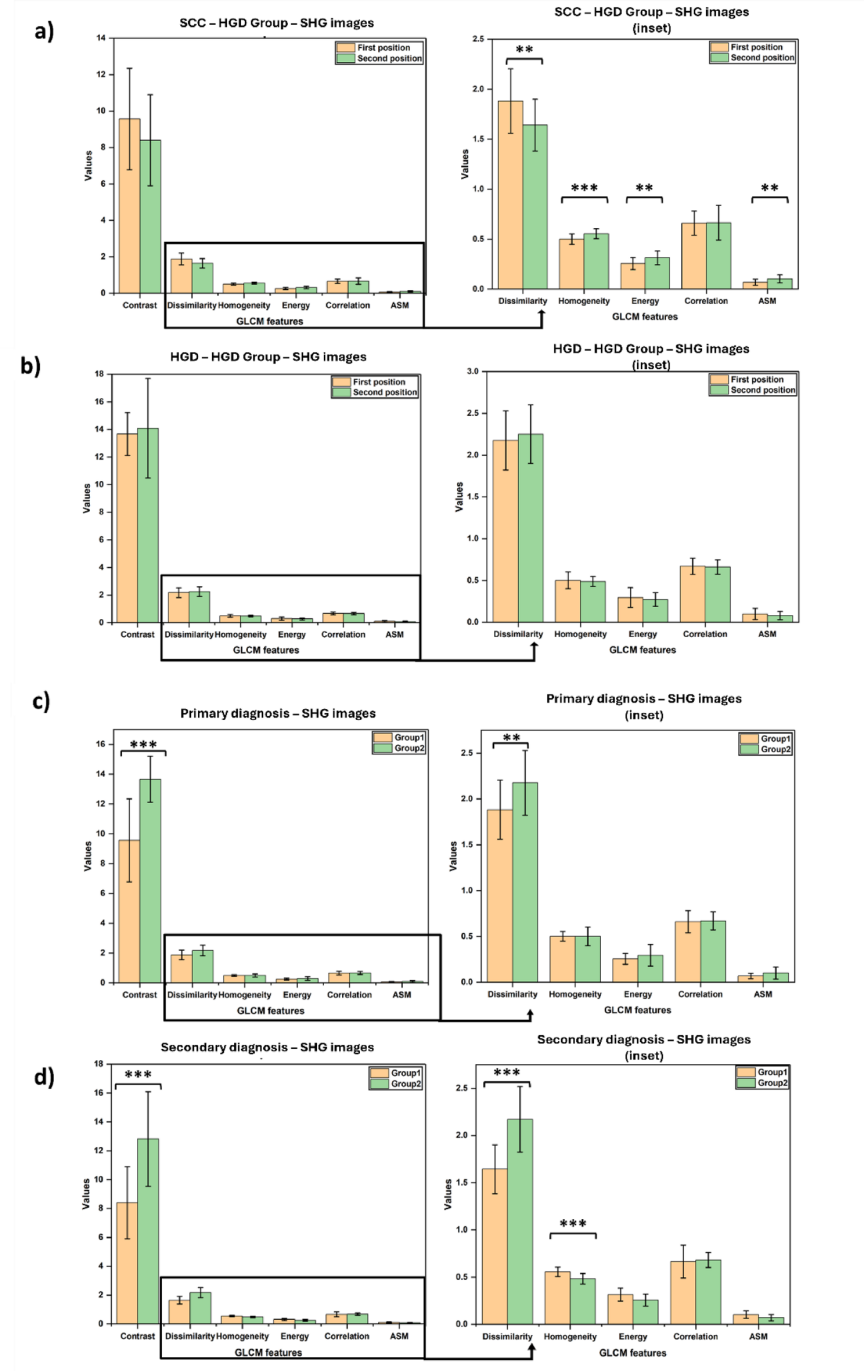
Comparison of the textural features of the SHG images of (a) Group 1, (b) Group 2, (c) First position, and (d) Second position. * indicates *p* < 0.05, ** indicates *p* < 0.01, and *** indicates *p* < 0.001. The error bars indicate the standard deviation. The right-hand panels are insets.

**Fig. 7:**
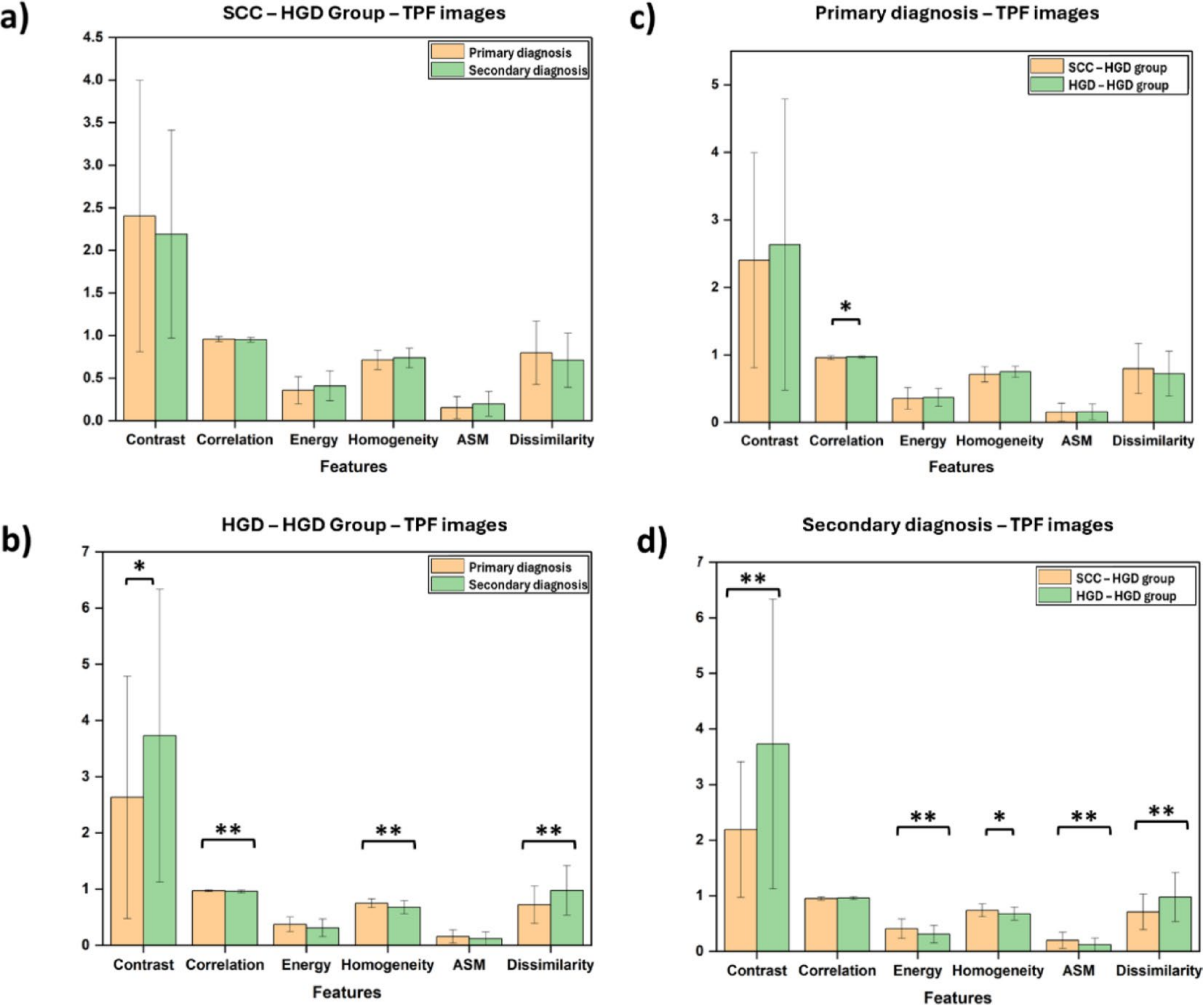
Comparison of textural features of TPF images (a) Group 1, (b) Group 2, (c) First position, and (d) Second position. * indicates *p* < 0.05, ** indicates *p* < 0.01, and *** indicates *p* < 0.001. The error bars indicate the standard deviation.

For the SHG images, within Group 1, the images from the first diagnosis of SCC had greater Contrast and Dissimilarity than did those from the metachronous HGD (Fig. 6(a)). This finding indicated that the complexity of the textural orientation of collagen was greater in the SCC than in HGD. SCC images in Group 1 were also found to have lower uniformity in terms of the textural pattern of collagen in the affected tissue. This was supported by the low textural feature values of the SCC images, such as Homogeneity, Energy and ASM, compared to those of the HGD images at the second diagnosis. On the other hand, the SHG images of the first diagnosis (HGD) in Group 2 had lower contrast values than those of the second diagnosis (HGD) (Fig. 6(b)),

Furthermore, a comparison was made between the two groups based on the diagnoses. A comparison of Group 1 and Group 2 based on the first diagnoses, i.e., SCC and HGD, revealed that the tissues were ambiguous based on their textural features (Fig. 6(c)). However, compared with those of SCC images, the Complexity and Dissimilarity of HGD images were found to be significantly different. On the other hand, a comparison of the second diagnoses of the two groups, i.e., both HGDs, revealed that the tissue matrix of dysplasia that progressed from SCC was less complex in texture and uniformity than that of Group 2 (Fig. 6(d)). The dysplastic lesions diagnosed in Group 2 after progression had a more complex texture, indicating greater changes in the ECM and collagen orientation of the tissue. The TPF images in all the groups were also analyzed in a similar manner and showed a similar trend to that seen in the SHG images (Fig. 7).

**Fig. 8:**
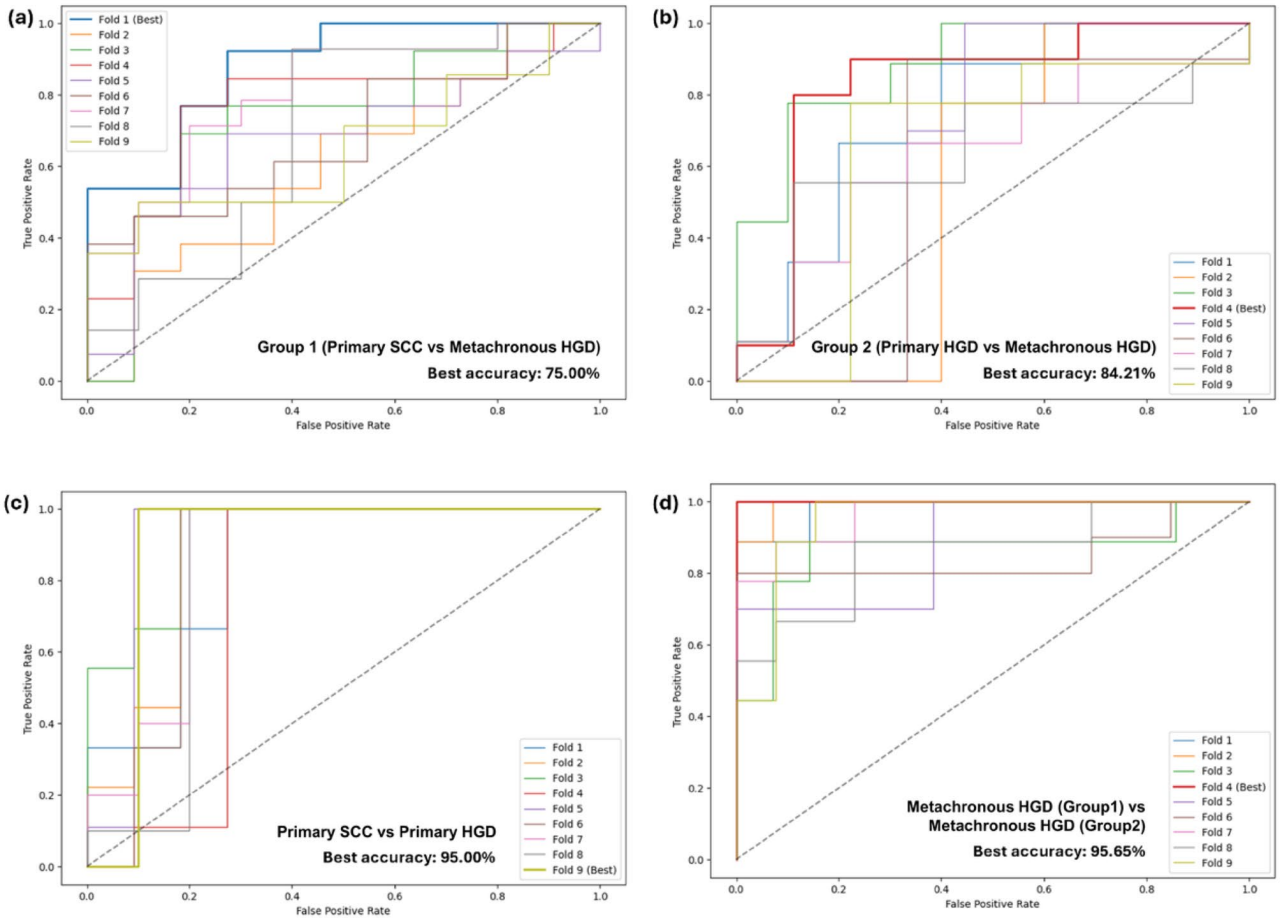
ROC-AUC curves for SVM classifiers of SHG image data (a) Group 1 (primary SCC vs. metachronous HGD), (b) Group 2 (primary HGD vs. metachronous HGD), (c) primary SCC vs. primary HGD and (d) metachronous HGD (Group 1) vs. metachronous HGD (Group 2).

**Fig. 9:**
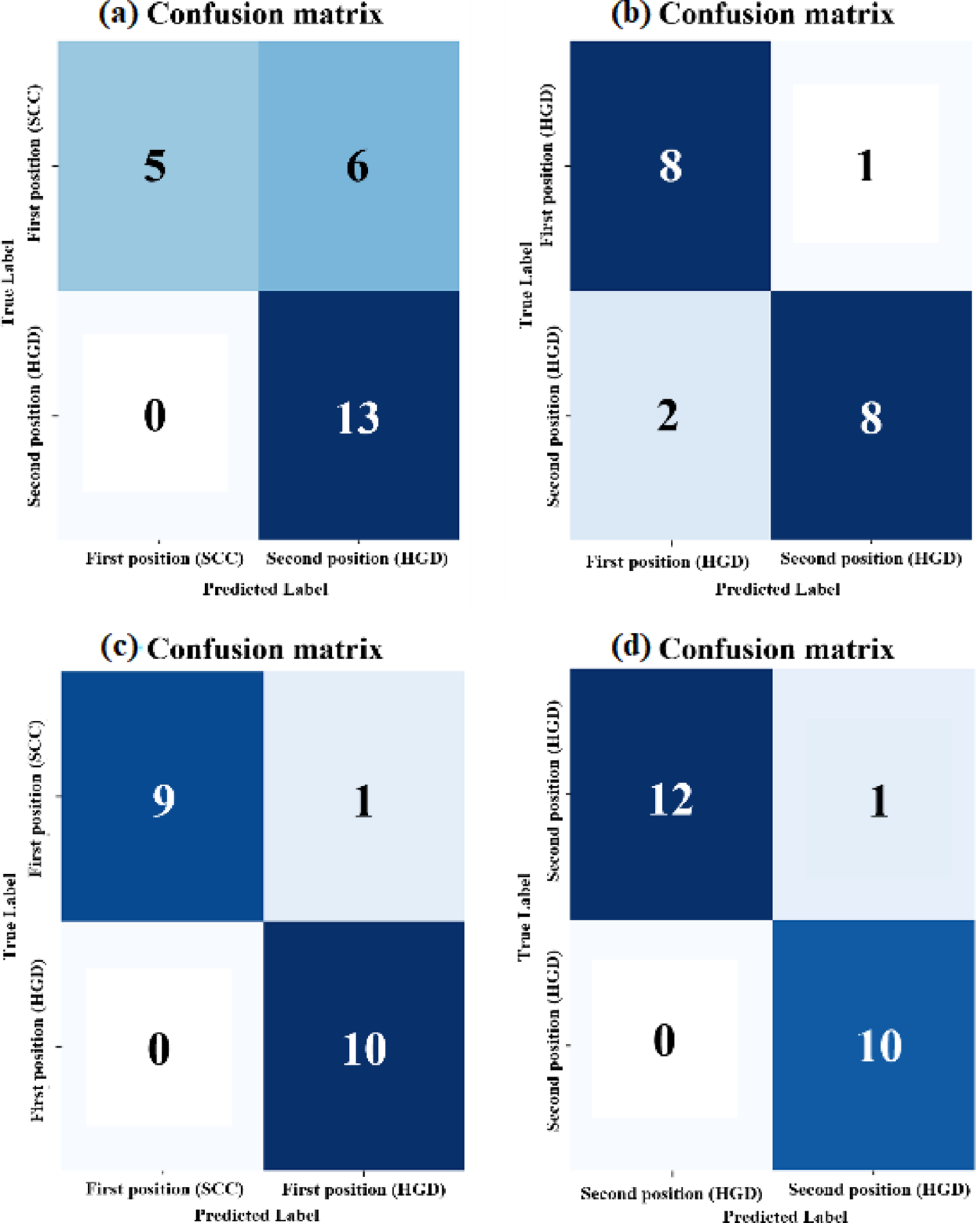
Confusion matrices of the best performing fold of the SVM classifiers of SHG image data (a) Group 1 (primary SCC vs metachronous HGD), (b) Group 2 (primary HGD vs metachronous HGD), (c) primary SCC vs primary HGD and (d) metachronous HGD (Group 1) vs metachronous HGD (Group 2).

The dataset of SHG image features contained 96 data points for primary SCC images, 120 data points for metachronous HGD images of Group 1, and 84 each for primary and metachronous HGD images of Group 2. Similarly, the dataset of TPF image features contained 192 data points for primary SCC images, 237 data points for metachronous HGD images of Group 1, and 128 each for primary and metachronous HGD images of Group 2. The textural feature obtained for each angle in which GLCM was computed and included as a data point in the study. Furthermore, the dataset was processed to eliminate blank spaces and extreme outliers which would have affected the study in a detrimental manner. Care was taken to ensure that these outliers would not affect the study. The stratified k-fold cross-validation results for texture-based SVM classifiers revealed distinct performance patterns across imaging modalities (SHG vs. TPF) and diagnostic groups. The SHG channel models (Figs. 8 and 9, and Table 1) demonstrated superior classification accuracy and AUC-ROC values compared to TPF-based models (Figs. S2 and S3, and Table S1), highlighting the critical role of collagen-specific textural features in discriminating esophageal cancer subtypes. In the Fig. 8, the AUC-ROC curves of each fold is shown for the models, and the best performing fold is highlighted. In case of SHG images, four SVM models were trained, the first one for the classification between primary SCC and metachronous HGD (Group 1), the next one for the classification between primary HGD and metachronous HGD (Group 2), one to distinguish between the first diagnoses SCC and HGD in the two groups, and another one to distinguish between the second diagnoses of HGD in both groups. The results of the ML models trained on SHG image data are represented in Table 1 and the same for TPF image data are shown in the supplementary material. The model for primary SCC vs. primary HGD shows remarkable capability, with an accuracy of 95.00% in differentiating between SCC and HGD which were both the first diagnoses of the groups of patients. The model for metachronous HGD of both groups was also able to distinguish between the second diagnoses of HGD for both groups of patients with an accuracy of 95.65%. This is an interesting find, for it shows that there is a fundamental difference in the development and progression of esophageal cancer in both groups of patients. In case of TPF images, again, two models were trained in a similar manner to that of the SHG images. The ML models of TPF image data are found to have lower distinguishing capabilities (Figs. S2 and S3, and Table S1 in the supplementary material) compared to that of the models trained with SHG data.

**Table 1:** SVM models and their corresponding accuracies for classification.

SVM Model name		Best fold accuracy	Average accuracy
SHG image data	Group 1 (primary SCC vs. metachronous HGD)	75.00%	71.63%
	Group 2 (primary HGD vs. metachronous HGD)	84.21%	67.94%
	primary SCC vs. primary HGD	95.00%	86.13%
	metachronous HGD (Group 1) vs. metachronous HGD (Group 2)	95.65%	92.23%

## Discussion

### Visualization of the morphological structure of esophageal tissues

In paraffin-embedded tissue sections, fluorescence changes rapidly after cell death and fixation. Additionally, the fluorescent molecules present in the tissue are further reduced by processing in alcohol and xylene, which removes many fluorophores. Therefore, the main focus of TPF imaging is to visualize the autofluorescent and fluorescent molecules (from H&E staining) on extracellular components and tissue architectures in SCC and HGD rather than on the size and organization of cell nuclei using excitable DNA labels^34,35^. On the other hand, the weak but detectable SHG signal originates primarily from extracellular collagen, which remains relatively stable during slide preparation. While TPF signals capture various tissue structures, they show no crosstalk with collagen SHG. This specificity makes SHG imaging a unique and reliable method for visualizing the structural organization of the ECM in esophageal tissues.

In the human esophagus, which extends from the inner lumen to the outer adventitia, the tissue is stratified into several layers: the keratinized layer, mucosa, submucosa, muscularis propria, and adventitia. Our study specifically targets the early onset of high-grade dysplasia, which was dissected using endoscopic submucosal dissection and primarily encompasses the mucosa and submucosa tissues. The mucosal layer is further subdivided into three distinct zones: the epithelium, lamina propria (LP), and muscularis mucosae (MM), as illustrated in Fig. 2(a). The epithelial layer is characterized by squamous cells that proliferate and ascend, continuously renewing the epithelial lining. The lamina propria, a slender layer of connective tissue, bridges the epithelium with the smooth muscle cells of the muscularis mucosae. It provides an essential blood supply to the epithelium and neural connections that stimulate the underlying muscularis mucosae. The muscularis mucosae plays a crucial role in stretching and contracting the mucosa, aiding in the formation of a mucosal plug or a one-way valve during swallowing.

Figures 2, 3, 4 and 5 show that collagen signals predominantly appear in the connective tissues of the LP and submucosa but not in the epithelium or MM. For instance, in Fig. 2(c), as dysplasia originates in the epithelium and gradually evolves into squamous cell carcinoma invading the submucosa, alterations in collagen within the LP can yield crucial insights into the current state of the corresponding HGD or SCC above it. To ensure accurate GLCM analysis, we excluded artifact signals from dye accumulation at section edges. These artifacts produce broadband emission in both spectral channels presumably due to photodamage. From these images, we found that the tissue morphology all contained layer structures, as indicated by Fig. 2(a), and that the ECM contained partially oriented collagen fibers (shown by SHG signals) nearly parallel to the epithelium. As these characteristics are visually similar, it is difficult to distinguish between the four types of tissues. To reveal the structural differences embedded in these tissues, ML algorithms, as demonstrated in the following sections, were used.

Recent studies in label-free computational histology have demonstrated that nonlinear optical imaging techniques integrated with AI/ML can provide diagnostic accuracy comparable to conventional H&E-based pathology. Studies utilizing SHG, TPF, and FLIM have shown that biologically significant information can be extracted without H&E stained images^36,37]]^. Motivated by these findings, our study focuses on two-photon image analysis as a standalone modality, aiming to capture subtle SHG and TPF extracellular matrix alterations and tissue architecture changes characteristic of dysplastic progression. Rather than using H&E-based classifiers as a baseline, we emphasize the unique textural and structural insights provided by two-photon microscopy to support the development of future label-free diagnostic strategies.

### GLCM analysis based classification using SVM

During ESCC progression, tumor cells consume collagen fibers, leading to disorientation of collagen and elastic fibers in the submucosa^38,39^. Previously, our group used the ratio of TPF to SHG and forward to backward (F/B) SHG from single collagen fibers for further characterizing ESCC cancer stages^10^. However, little is known about the differences in the ECM status between the primary tumor site and metachronous site. Here, we investigated the different morphological features of collagen fibers in the ECM between primary HGD with metachronous HGD and primary SCC with metachronous HGD groups and evaluated whether these differences can be used as biomarkers for early cancer detection. Currently, there are multiple approaches for the analysis of collagen in the ECM, such as curvelet transform and texture analysis. There are also open-source software tools such as ImageJ plug-ins, and standalone applications CytoSpectre, and CurveAlign for the analysis of collagen fibers. FibrilTool, an ImageJ plug-in, calculates the main fiber orientation in the ROIs of tissue images using the local nematic tensor concept from liquid crystal physics and the first derivative^40^. However, manual input is needed for evaluation and ROI selection, making high-throughput processing time-consuming^41,42^. Orientation J, another ImageJ plug-in, calculates pixel orientation and anisotropy with a structure tensor from the first derivative and Gaussian weighting, identifying fiber pixels and assigning weights for orientation^43^. However, the tool necessitates manual ROI annotation, thus rendering it non-ideal for larger image datasets^44,45^. CytoSpectre, a standalone software tool, extracts orientation and anisotropy in a window using Fourier transform analysis. However, obtaining localized fiber data from individual fibers in the ROIs is challenging^46,47^. CurveAlign, another standalone tool, is a comprehensive platform for quantifying fibrillar collagen with two fiber analysis methods: CT-FIRE and curvelet fiber representation. However, it was found that the extraction of entire fibers, particularly those with curved shapes and varying intensities along their length, is challenging through the tool, thus resulting in the tracking of long curved fibers ending up with only a few segments along the fiber propagation direction. More importantly, the fiber tracking algorithm in CurveAlign is computationally intensive, leading to longer processing times. This is a challenge when working with very large images or larger image datasets^15,48^. In contrast to the aforementioned software tools, the GLCM textural analysis method is efficient and computationally less intensive. Furthermore, the textural features extracted from the GLCM algorithm are also useful for training ML algorithms to differentiate between the different pathological states of a tissue^49,50^. Thus, the acquired images were analyzed using the GLCM method to quantitatively classify SCC-HGD and HGD-HGD based on the first and second diagnostic results and determine the origin of the lesions that induce variations in the structure and organization of collagen fibers during diseased tissue progression in patients with esophageal cancer.

The textural features from SHG signals highlight collagen disorganization in SCC when compared to HGD, with increase in complexity and lower uniformity. The changes observed between the first and second diagnoses also suggest progressive ECM remodeling during disease progression, indicating a trend toward increased tissue complexity from the first diagnosis to the second diagnosis. This is supported by low values of Contrast and Dissimilarity for images from the first diagnosis and higher values for Energy and ASM. This increase in tissue complexity is indicative of greater changes in the orientation of collagen fibers in the ECM of the tissue, which is in turn indicative of disease progression between the first and second diagnosis of Group 2 patients^50^.

The comparison made between the two groups revealed the textural ambiguity (Fig. 6(c)). However, compared with those of SCC images, the Complexity and Dissimilarity of HGD images were found to be significantly different. This ambiguity in the uniformity of the textural features of the images could be due to the inherent heterogeneity and complexity of the SCC and HGD tissues in terms of their morphology and organization of the ECM, thus making it challenging to extract consistent textural feature data from the images. On the other hand, HGDs from Group 1, revealed that the tissue matrix of dysplasia that progressed from SCC was less complex in texture and uniformity than that of Group 2 (Fig. 6(d)). The dysplastic lesions diagnosed in Group 2 after progression had a more complex texture, indicating greater changes in the ECM and collagen orientation of the tissue due to progression of the disease. This may be indicative of the differences in the pathologies of the two dysplasias between the groups based on their origin. In addition, it can be hypothesized that there is a therapeutic effect in Group 1 after chemotherapy or radiotherapy, which makes the collagen fibers healthier (i.e., less complex and more uniform) than those in Group 2^51,52^.

The TPF image analysis that indicated of similar changes in the other components of the ECM and other layers of the esophageal tissue^53^demonstrated a concerning high standard deviation in the Contrast feature. However, Contrast, as derived from the GLCM, measures the intensity difference between neighboring pixels, with higher values indicating more pronounced texture variations. The observed variability in Contrast can be attributed to the differences in collagen fiber arrangement, tumor-associated ECM remodeling, and inter-patient variability.

The integration of GLCM textural analysis with SVM classification provides a robust computational framework for quantifying collagen disorganization in SCC and HGD. Our results demonstrate that GLCM-derived features, particularly Contrast, Energy, and Dissimilarity, effectively captures progressive ECM remodelling during malignant transformation, aligning with histopathological evidence of tumour-induced collagen degradation*.*

The features obtained from the GLCM feature extraction algorithm were used to train the SVM classifier. Our analytical approach focused on group-wise comparison using stratified cross-validation to address our primary aim, which is to understand the distinct pathological progression patterns between primary SCC progressing to HGD (Group 1) versus primary HGD progressing to metachronous HGD (Group 2). This comparison specifically investigates whether the origin of dysplasia influences its subsequent characteristics and progression patterns.

The classification accuracy of the Group 2 SVM model (84%) vs. Group 1’s (75%) holds critical clinical implications for distinguishing between primary and metachronous HGD. While Group 1 (primary SCC with metachronous HGD) involves more overt stromal disruption during malignant progression, the accuracy in Group 2 (primary HGD with metachronous HGD) suggests that even subtle collagen reorganization in precancerous lesions can be detected through GLCM-SVM analysis. This aligns with the clinical challenge of monitoring HGD progression, where early ECM changes often precede histologically evident malignancy. The model’s ability to resolve textural differences between primary and metachronous HGD indicates its utility in identifying stromal remodelling that may signal imminent neoplastic transformation—a capability critical for guiding personalized surveillance intervals in Barrett’s esophagus patients^54^. From a therapeutic standpoint, the 84% accuracy in Group 2 underscores the potential of texture-based biomarkers to augment conventional dysplasia grading, which suffers from interobserver variability. By quantifying progressive collagen disorganization, this approach has the potential to aid in the stratification of HGD patients at highest risk of occult carcinoma, enabling timely endoscopic intervention. However, to get to this stage, it would require further investigations and correlation with multiple clinical parameters to consolidate the results.

Furthermore, the exceptional performance of both SVM models—95% accuracy for primary SCC vs. HGD classification and 95.65% accuracy for metachronous HGD subtype discrimination—underscores the diagnostic potential of collagen textural biomarkers in esophageal cancer management. These results reflect fundamental differences in ECM organization between distinct pathological states and their progression trajectories. TPF-based classifiers performed consistently lower across both groups. Higher ROC-AUC in Group 1 indicates more pronounced ECM remodeling during SCC progression.

From a clinical perspective, the findings support the potential of two-photon imaging combined with SVM algorithm for early cancer detection. However, the relatively lower classification accuracy for metachronous HGD highlights the need for further improvements. Future work should focus on increasing the dataset size, integrating additional imaging modalities, and employing more advanced feature extraction methods to refine the classification of HGD progression. While our current validation is limited to our dataset, the methodology demonstrates potential for broader application, particularly in monitoring disease progression and potentially predicting outcomes. However, we also acknowledge the requirement of further validation on larger, independent cohorts before clinical implementation. Future studies with larger cohorts should incorporate more feature extraction methods for a more robust classification, to establish broader generalizability. Additionally, incorporating temporal tracking of textural changes could enhance our understanding of disease progression patterns and improve predictive capabilities.

Unlike prior studies that focused primarily on qualitative descriptive imaging of esophageal cancer using two-photon microscopy^55^, our work integrates quantitative ECM texture analysis using GLCM features with SVM to classify specific pathological transitions within and across patients. This dual-group framework (SCC-HGD and HGD-HGD) enables direct evaluation and monitoring of disease progression stages. Furthermore, by demonstrating high classification accuracy, particularly for SHG signal-based features from collagen, this novel approach shows the potential for using these features as objective biomarkers of ECM remodeling. This provides automated tissue diagnosis that can assist pathologists in early disease detection, ultimately aiding clinical practice.

## Conclusion

We integrated two-photon imaging with ML algorithms to complement current clinical methods for characterizing different pathological stages and understanding the pathogenic factors involved in the progression of esophageal cancer. To analyze the structural differences between Group 1 and Group 2, we conducted four comparisons by evaluating their GLCM texture features and corresponding statistics. The SHG-based classifiers demonstrated 75%, 84.21%, 95%, and 95.65% for Group 1 (primary SCC vs. metachronous HGD), Group 2 (primary HGD vs. metachronous HGD), primary SCC vs. primary HGD, and metachronous HGD (Group 1) vs. metachronous HGD (Group 2) respectively. The results demonstrate that the complexity of the textural orientation of collagen fibers is the dominant factor in the changes observed in the ECM structure and accompanies cancer progression. These features can serve not only as biometric features for distinguishing between different pathological tissues but also as indicators for predicting the occurrence of early cancer, highlighting their significant potential for clinical research. By bridging two-photon microscopy with automated image analysis, this approach can be further validated across other cancer types to identify specific structural factors for early diagnosis and establish a comprehensive database for biomedical research.

## Supplementary Information

Below is the link to the electronic supplementary material.

Supplementary Material 1

## Data Availability

The datasets used and/or analyzed during the current study available from the corresponding author on reasonable request.
