# Supplementary material for: Quantitative image analysis of the extracellular matrix of esophageal squamous cell carcinoma and high grade dysplasia via two-photon microscopy
Source: Sci Rep. 2025 Aug 7;15:28943. doi: 10.1038/s41598-025-13910-7 (PMC12332014; doi:10.1038/s41598-025-13910-7)
Supplement: Supplementary file 1 — Supplementary Material 1 [file 41598_2025_13910_MOESM1_ESM.docx]

**Quantitative image analysis of the extracellular matrix of esophageal squamous cell carcinoma and high-grade dysplasia via two-photon microscopy**

Kausalya Neelavara Makkithaya^1,†^, Wei-Chung Chen^2,†^, Chun-Chieh Wu^3^, Ming-Chi Chen^4^, Wei-Hsun Wang^4^, Jackson Rodrigues^5^, Ming-Tsang Wu^6^, Nirmal Mazumder^1,*^, I-Chen Wu^2,7*^, and Guan-Yu Zhuo^5,*^

^1^Department of Biophysics, Manipal School of Life Sciences, Manipal Academy of Higher Education, Manipal 576104, India

^2^Division of Gastroenterology, Kaohsiung Medical University Hospital, Kaohsiung Medical University, Kaohsiung 807, Taiwan

^3^Department of Pathology, Kaohsiung Medical University Hospital, Kaohsiung Medical University, Kaohsiung 807, Taiwan

^4^Institute of Translational Medicine and New Drug Development, China Medical University, Taichung 404328, Taiwan

^5^Institute of Biophotonics, National Yang Ming Chiao Tung University, Taipei 11221, Taiwan

^6^Department of Family Medicine, Kaohsiung Medical University Hospital, Kaohsiung Medical University, Kaohsiung 807, Taiwan

^7^Center for Cancer Research; Center for Liquid Biopsy and Cohort Research, Kaohsiung Medical University, Kaohsiung 807, Taiwan

^†^Contributed equally to this work

^*^Authors to whom correspondence should be addressed: [nirmal.mazumder@manipal.edu](mailto:nirmal.mazumder@manipal.edu); [minicawu@gmail.com](mailto:minicawu@gmail.com); [zhuo0929@gmail.com](mailto:zhuo0929@gmail.com)


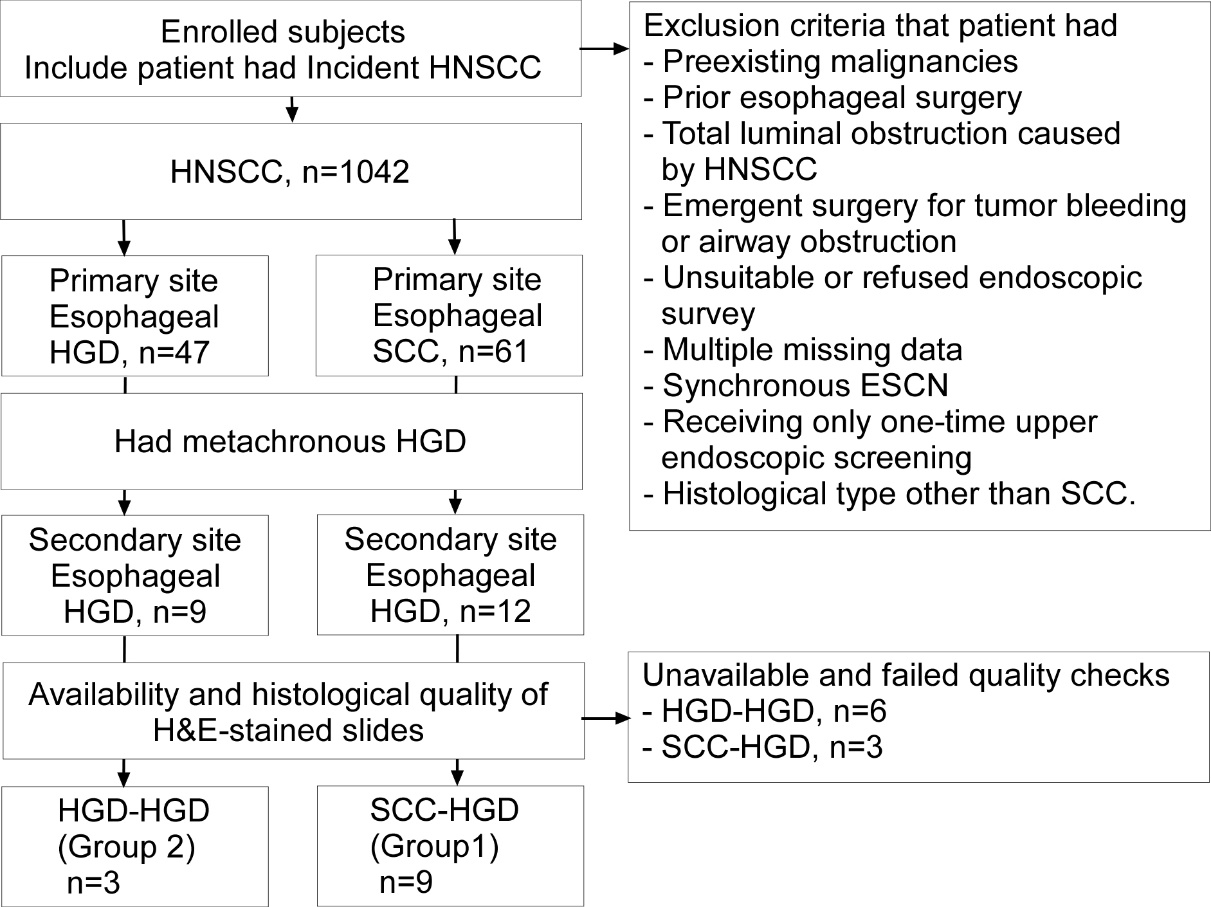


**Fig. S1.** Flowchart of patient selection and grouping for histological evaluation of metachronous esophageal HGD in individuals with incident HNSCC. HGD: High-Grade Dysplasia; HNSCC: Head and Neck Squamous Cell Carcinoma.

**GLCM analysis-based TPF image classification using SVM**

The SVM model was trained with the GLCM features obtained from TPF imaging for further comparison of textural data obtained from Esophageal cancer images of the two modalities. Furthermore, an area under the curve of the receiver operating characteristic (AUC-ROC) curve was plotted for each classifier, wherein the AUC-ROC curve is plotted for each fold and the fold with the best performance is highlighted. The corresponding confusion matrix for the best performing fold in the model is also plotted along with AUC as shown in Fig. S2. The corresponding confusion matrix showing the performance of the trained model is shown in Fig. S3.

**
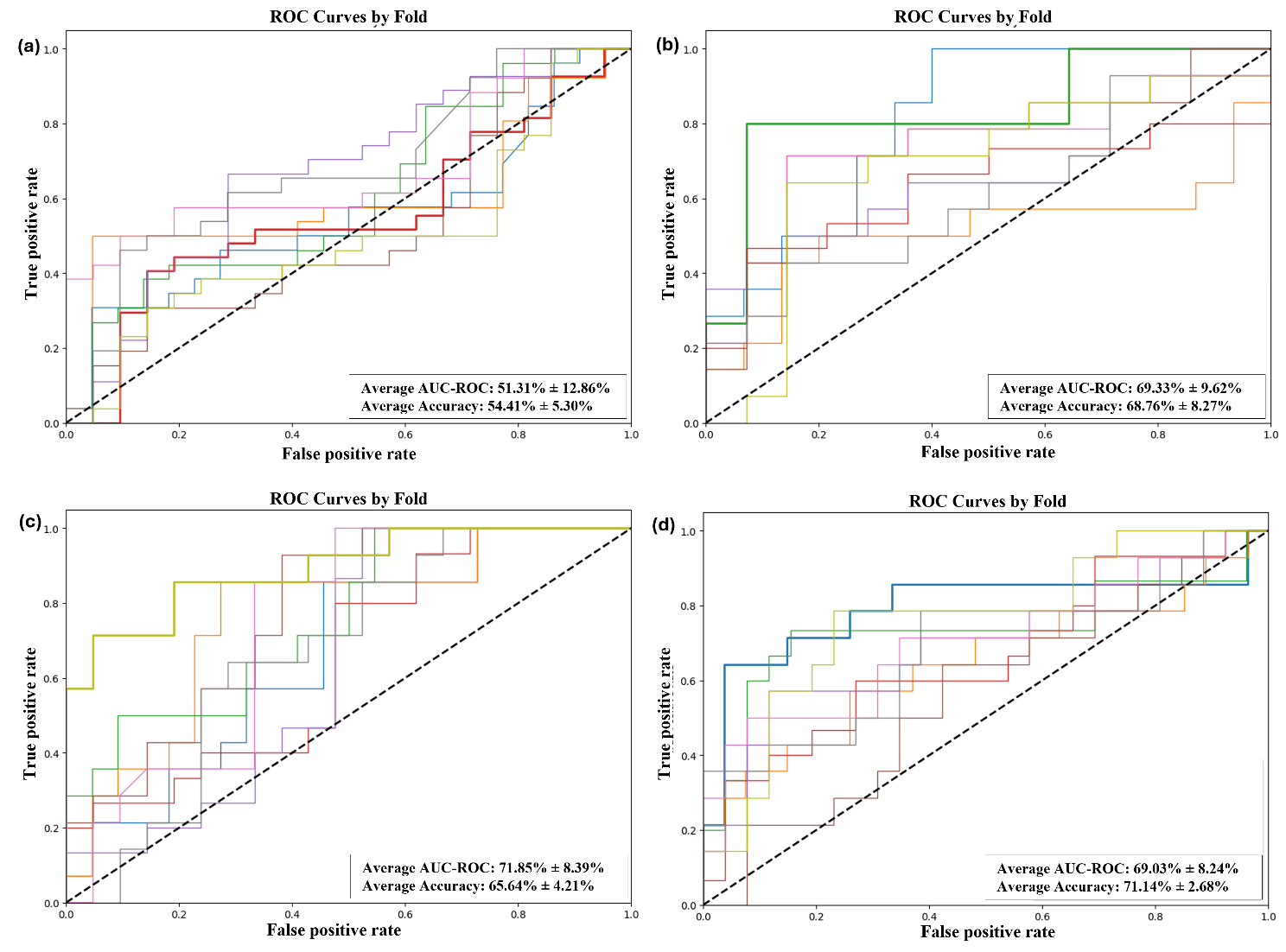
**

**Fig. S2.** ROC-AUC curves for SVM classifiers of TPF image data (a) Group 1 (primary SCC vs metachronous HGD), (b) Group 2 (primary HGD vs metachronous HGD), (c) primary SCC vs primary HGD and (d) metachronous HGD (Group 1) vs metachronous HGD (Group 2).


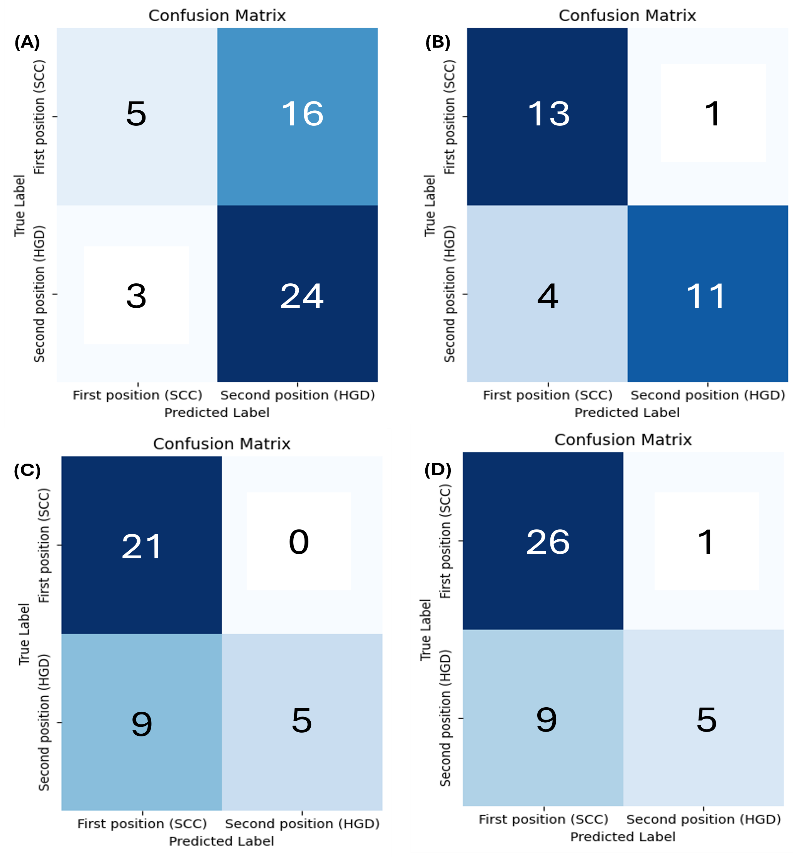


**Fig. S3.** Confusion matrices of the best performing fold of the SVM classifiers of TPF image data (A) Group 1 (primary SCC vs metachronous HGD), (B) Group 2 (primary HGD vs metachronous HGD), (C) primary SCC vs primary HGD and (D) metachronous HGD (Group 1) vs metachronous HGD (Group 2).

Table S1: SVM models and their corresponding accuracies for classification:

| TPF image data | Group 1 (primary SCC vs metachronous HGD) | 60.72% | 51.31% |
| --- | --- | --- | --- |
|  | Group 2 (primary HGD vs metachronous HGD) | 82.76% | 69.33% |
|  | primary SCC vs primary HGD | 74.29% | 71.85% |
|  | metachronous HGD (Group 1) vs metachronous HGD (Group 2) | 75.61% | 69.03% |

The classification accuracy as shown in Table S1 varies across comparisons of SCC vs HGD using different label orders. Group 1 showed the lowest accuracy at 60.7%, due to misclassifications with SCC. Accuracy improved in Group 1 to 82.76% and remains moderate in primary SCC vs primary HGD and metachronous HGD (Group 1) vs metachronous HGD (Group 2) at 74.29% and 75.61%, respectively. In the current study, this variation highlights the challenge of distinguishing SCC and HGD by TPF image analysis.
